# Utility of Circulating Tumor DNA for Detection and Monitoring of Endometrial Cancer Recurrence and Progression

**DOI:** 10.3390/cancers12082231

**Published:** 2020-08-10

**Authors:** Esther L. Moss, Diviya N. Gorsia, Anna Collins, Pavandeep Sandhu, Nalini Foreman, Anupama Gore, Joey Wood, Christopher Kent, Lee Silcock, David S. Guttery

**Affiliations:** 1 Leicester Cancer Research Centre, College of Life Sciences, University of Leicester, Leicester LE2 7LX, UK; dg251@le.ac.uk (D.N.G.); ac763@le.ac.uk (A.C.); pps8@le.ac.uk (P.S.); nm14@le.ac.uk (N.F.); 2 Department of Gynaecological Oncology, University Hospitals of Leicester NHS Trust, Leicester General Hospital, Leicester LE5 4PW, UK; anupama.gore@uhl-tr.nhs.uk (A.G.); Joey.Wood@uhl-tr.nhs.uk (J.W.); Christopher.Kent@uhl-tr.nhs.uk (C.K.); 3 Nonacus Limited, Birmingham Research Park, Birmingham B15 2SQ, UK; lee.silcock@nonacus.com

**Keywords:** endometrial cancer, circulating tumor DNA, digital droplet PCR, ion torrent, whole exome sequencing

## Abstract

Despite the increasing incidence of endometrial cancer (EC) worldwide and the poor overall survival of patients who recur, no reliable biomarker exists for detecting and monitoring EC recurrence and progression during routine follow-up. Circulating tumor DNA (ctDNA) is a sensitive method for monitoring cancer activity and stratifying patients that are likely to respond to therapy. As a pilot study, we investigated the utility of ctDNA for detecting and monitoring EC recurrence and progression in 13 patients, using targeted next-generation sequencing (tNGS) and personalized ctDNA assays. Using tNGS, at least one somatic mutation at a variant allele frequency (VAF) > 20% was detected in 69% (9/13) of patient tumors. The four patients with no detectable tumor mutations at >20% VAF were whole exome sequenced, with all four harboring mutations in genes not analyzed by tNGS. Analysis of matched and longitudinal plasma DNA revealed earlier detection of EC recurrence and progression and dynamic kinetics of ctDNA levels reflecting treatment response. We also detected acquired high microsatellite instability (MSI-H) in ctDNA from one patient whose primary tumor was MSI stable. Our study suggests that ctDNA analysis could become a useful biomarker for early detection and monitoring of EC recurrence. However, further research is needed to confirm these findings and to explore their potential implications for patient management.

## 1. Introduction

Endometrial cancer (EC) is the second most common female cancer in the US, with over 60,000 women diagnosed each year [1]. In contrast to the high survival rate in patients with primary disease, the prognosis upon recurrence is poor, with a 5-year survival rate in patients with distant recurrence of 17% [2,3], and the majority of EC recurrences in high-risk cases developing distant metastases within the first 3 years post-treatment [4]. There is no specific blood protein biomarker that is recommended for use following an EC diagnosis; instead, regular clinical examination is advised with imaging. The ovarian cancer markers CA125 and HE4 have been shown to significantly correlate with prognosis; however, their detection rates in metastatic disease are low—54% and 75% respectively—and even lower with local recurrence: 39% and 16% [5]. Hence, more effective biomarkers are required that can detect EC recurrence and disease progression earlier, as well as reliably reflecting the underlying kinetics of the disease.

The liquid biopsy is now recognized as a less-invasive method for early cancer detection and monitoring tumor heterogeneity and evolution. Major clinical trials are currently underway assessing the utility of both circulating tumor cells (CTCs) and circulating tumor DNA (ctDNA) for monitoring disease kinetics, with both illustrating complementary sensitivity and specificity while offering their own unique strengths regarding prognosis (especially CTCs) and early detection of relapse (ctDNA) [6,7,8,9,10]. There are now numerous methods for detecting both CTCs and ctDNA. The Menorini CellSearch^TM^ system is the only technology available to date that is FDA-approved and used in clinical trials [11]; however, the CellSearch system relies on expression of the surface marker EpCAM for CTC enrichment and many CTCs (including those undergoing epithelial-to-mesenchymal transition and therefore do not express EpCAM), as well as small CTCs and CTC clusters, are missed [12]. As a result, other technologies (such as Parsortex and RareCyte) offer alternative methods for CTC enrichment based on cell size and density [13].

Cell-free DNA (cfDNA) can be extracted from plasma and the tumor-derived fraction of cfDNA, known as circulating tumor DNA (ctDNA) is rapidly becoming a quick and sensitive biomarker for tracking minimal residual disease and accurately reflecting therapeutic response in patients with actionable hotspot mutations [14,15], chromosomal rearrangements [16], and gene amplifications [17,18]. As with CTCs, numerous methods are available for total cfDNA extraction from both serum and plasma, with studies illustrating that several pre-analytical factors affect yield and downstream analysis post-venepuncture, including the blood tube used, number of centrifugation steps, centrifugal speed, cfDNA extraction method, and ctDNA detection methods [19,20,21]. Methods employed for detection of ctDNA depend upon the question being asked, with whole genome/exome next generation sequencing (NGS) being performed to obtain a global view of genomic changes associated with disease progression [22,23] (however, this is hampered by low sensitivity), targeted sequencing to obtain higher sensitivity (1 ctDNA molecule in 1000 cfDNA molecules) in a smaller number of genes [6,15,17,24,25], or digital droplet PCR (ddPCR) (usually analysis of one or two mutations to a sensitivity of one in 100,000) to detect early stage disease [14,26]. To date, only a handful of ctDNA tests have been approved by the U.S Food and Drug Administration (FDA) for use in metastatic and locally advanced NSCLC, and HR+, HER2-negative, advanced breast cancer in progression on or after endocrine therapy. The cobas^®^ *EGFR* Mutation v2 was the first to be approved using both tissue and plasma from patients with non-small cell lung cancer (NSCLC) that identifies 42 mutations in exons 18, 19, 20, and 21 of the epidermal growth factor receptor (*EGFR*) gene, including the T790M resistant mutation [27]. The Qiagen Therascreen^®^ PIK3CA RGQ PCR Kit for the detection of 11 mutations in the *PIK3CA* gene in ctDNA and/or tumor tissue received U.S. regulatory approval for use in guiding treatment decisions in patients with breast cancer undergoing treatment with Alpelisib [28]. More recently, the FDA has granted Natera’s Signatera personalized NGS test breakthrough device designation for use in the post-surgical detection and quantification of ctDNA in the blood of patients previously diagnosed with a number of certain types of cancer, and in combination with certain drugs [6,29,30]. In addition, other companion NGS liquid biopsy tests that are currently used in clinical trials include Guardant360 [31], Foundation One Liquid [32], and the MSK-IMPACT assay [33].

Although many studies have shown the ability of ctDNA for tracking and monitoring disease in cancers, such as lung [29], breast [6], colon [30], and prostate cancer [34], few studies have highlighted its promise in EC. CtDNA has been detected in 18% of primary ECs using next-generation sequencing [24], as well as showing elevated levels 6 months before a rise in CA125 or radiological evidence of recurrence on CT imaging in gynecological cancers [35]. More recent studies using ddPCR have shown that ctDNA can be detected in 40% of primary ECs [26]. However, to date, evidence is sparse highlighting the utility of ctDNA for detecting EC recurrence and monitoring treatment response during routine follow-up. Here, we used a combination of methods including targeted next-generation sequencing (tNGS), personalized ctDNA NGS analysis, and digital droplet PCR (ddPCR), with the primary objective of detecting and monitoring EC recurrence and therapeutic response.

## 2. Methods

The study was approved by the Wales Research Ethics Committee 7 (17/WA/0342, approval date 19 April 2017). All patients gave written informed consent prior to participation for use of their blood and tissue samples. Patients were recruited to the study if they were over the age of 18 and attending hospital follow-up appointments after an EC diagnosis, either after completion of primary treatment or after disease relapse. The reporting census date for this study was 15 September 2019.

### 2.1. Extraction and Quantitation of DNA

Blood sampling and processing was performed as previously described [36]. Efficient pre-analytical handling of blood samples post-venepuncture and prior to cfDNA extraction is a crucial step in ensuring optimal downstream analysis [8,19]. Although there is currently no consensus on pre-analytical protocols, we have developed a well-established method for ensuring contamination of plasma with germline DNA is minimalized as much as possible [36,37]. Briefly, 20 mL of blood was obtained into EDTA K2 tubes and centrifuged at 1000× *g* for 10 min at 4 °C within 2 h post-venepunture to circumvent white blood cell (WBC) lysis prior to extraction. Plasma was further centrifuged at 2000× *g* for 10 min at 4 °C to remove cell debris and stored at −80 °C until required, along with 400 μL of buffy coat for germline DNA extraction. To minimalize freeze-thaw effects [38,39], plasma was only thawed once prior to extraction. Prior to cfDNA extraction, plasma underwent an additional centrifugation step at 1000× *g* to ensure complete removal of contaminating cells, which we have shown previously to be an essential step in minimizing contamination of cfDNA with germline DNA [36,37]. Total cell-free DNA (cfDNA) was isolated from at least 3 mL of plasma using the QIAamp Circulating Nucleic Acid Kit (Qiagen, GmbH, Hilden, Germany) or using the MagMAX Cell-Free DNA Isolation Kit (Thermofisher, Waltham, MA, USA) in combination with the Kingfisher Flex System (Thermofisher, Waltham, MA, USA) in an automated manner, according to manufacturer’s instructions. These methods were used as they are optimized for extraction of shorter cfDNA fragments (i.e., between 50 bp–200 bp), which are known to harbor the vast majority of ctDNA [40], and thereby ensuring contamination with germline DNA is minimalized. In-house analysis of numerous cfDNA samples using TapeStation has shown that the majority of cfDNA fragments are indeed within the 50–200 bp range (with the median being ~167 bp, associating cfDNA with mononucleosomes [41]), showing, again, that contamination with germline DNA is minimalized (expected range for germline DNA is >10,000 bp). This has also been shown by others [42,43,44], confirming that robust pre-analytical handling of blood samples is a critical factor in cfDNA analysis [19,36,45]. Germline DNA was extracted from 200 μL of buffy coat using the QIAamp DNA Blood Mini kit (Qiagen), according to manufacturer’s instructions. cfDNA levels (ng/mL) were converted to copies/mL plasma assuming 3.3 pg DNA per haploid genome. FFPE tumor DNA was extracted from 1.5 mm tissue cores using the Qiagen GeneRead Kit according to manufacturer’s instructions. Quantitation of total cfDNA, germline and tumor DNA was performed using qPCR, as described previously [36]. qPCR was used to determine DNA concentration, as this offers an important QC step regarding the amount of amplifiable DNA that is present in the sample, which is known to affect downstream analysis [46].

### 2.2. Targeted Next Generation Sequencing

Targeted NGS was performed on 20 ng of FFPE tumor DNA and lymphocyte DNA, and at least 5 ng of cfDNA (range 5–50 ng), using the Oncomine^TM^ Pan-cancer cfDNA assay (Thermofisher—52 genes covering >900 COSMIC mutations and 12 CNV regions). Libraries were prepared using the Ion Chef and sequenced on Ion 540 chips using the Ion S5 XL (ThermoFisher, Waltham, MA, USA), according to manufacturer’s instructions, to a minimum average read depth of at least 10,000×. Sequencing data was accessed through the Torrent Suite v5.6 and analyzed using Ion Reporter v5.6. For FFPE and lymphocyte DNA, the Oncomine TagSeq Pancan Tumor w1 workflow was used. For cfDNA, the Oncomine TagSeq Pancan Liquid Biopsy w1 workflow was used. Initially, targeted NGS using the Oncomine^TM^ Pan-Cancer cfDNA assay was conducted to identify the somatic mutations present in the primary tumor tissue in all cases; in two cases, additional biopsies from metastatic and recurrent disease were also sequenced.

### 2.3. Digital Droplet PCR

Droplet digital PCR (ddPCR) was performed to analyze the *TP53* p.Y220C mutation (assay numbers dHsaCP2500536 and dHsaCP2500537; Bio-Rad Laboratories) and *KRAS* p.G12D mutation (assay numbers dHSaCP2000001 and dHsaCP2000002), according to manufacturers’ instructions. Thermal cycling conditions were: 10-min hold at 95 °C, 40 cycles of 95 °C for 15 s, and then 55 °C for 60 s. Raw fluorescence amplitude was analyzed using the Quantasoft version 1.6.6.0320 software (Bio-Rad Laboratories, Hercules, CA, USA) [9].

### 2.4. Whole Exome Sequencing and Personalized ctDNA Sequencing

The Cell3 Target Whole exome kit (Nonacus Ltd., Birmingham, UK) and Illumina NextSeq500 (Illumina, San Diego, CA, USA) was used to perform whole-exome sequencing on 150–200 ng of tumor DNA from each FFPE primary tumor block and 50 ng of germline DNA at an average read depth of 200× for tumor DNA, and 50× for matched lymphocyte samples. Personalized ctDNA panels were developed for analysis of at least 2 SNVs per patient (Table S1), using at least 5 ng of cfDNA (range 16–25 ng). Libraries were prepared using the Cell3™ Target Custom Panel (Nonacus Ltd., Birmingham, UK) and sequenced using an Illumina MiSeq to a minimum depth of 30,000×.

### 2.5. Bioinformatic Analysis

Binary Alignment Map (BAM) files from whole exome sequencing or personalized ctDNA assays were prepared using an in-house pipeline to process sequencing output after demultiplexing. The BAM files were then aligned to human genome reference 38 (GRCh38). Somatic variants arising from whole exome sequencing (WES) were called using two variant callers, Platypus [47] and Mutect2 [48] whilst variants detected in cfDNA using the personalized cfDNA assay were called using solely Platypus. Variants were called using a minimum base quality and minimum mapping quality of 20, with a minimum of 1 read fitting these parameters for variants to be identified. Additionally, variants were filtered for those with an 11-base window around to the variant location to check for variants with a minimum quality <15. The output VCF files were annotated using ANNOVAR [49] to identify and annotate the subsets and variants. Microsatellite instability was analyzed using MSIsensor [50] for the detection of mononucleotide repeats at several genomic sites (BAT-25, BAT-26, NR-21, NR-24, and NR-27) in tumor alleles, as determined by a shift in ≥3 bp in each marker. MSI-high (MSI-H) tumors were defined as those with ≥2 unstable markers, whilst samples with 1 unstable markers were classified as MSI low tumors, and those with 0 unstable markers were classified as microsatellite stable (MSS).

## 3. Results

In total, a cohort of 13 cases of mixed risk and mixed histology were analyzed. At the reporting census date, six patients had recurred/progressed, three had died (two of their disease and one of a non-EC cause) and four patients were clinically well, with no clinical or radiological sign of recurrence/progression (Table 1).

### 3.1. Sequencing of Primary Tumors

Initially, targeted NGS using the Oncomine^TM^ Pan-Cancer cfDNA assay was conducted to identify the somatic mutations present in the primary tumor tissue in all cases; in two cases additional biopsies from metastatic and recurrent disease were also sequenced for cases 5 and 9, respectively. At least one somatic hotspot mutation at an arbitrary cut-off of ≥20% VAF was detected in 9/13 (69%) patient tumors that was not present in the matched lymphocytes (Table S2). In the other four cases, matched tumor and normal samples were sequenced using a whole exome (WES) approach, then used to guide development of a custom targeted cfDNA panel (Table S1). For details of the mutations detected in each patient and selected for custom ctDNA panel design, see Table S2. Two of the patients sequenced using WES (patients 1 and 2) were found to have an unusually high number of single nucleotide variants (SNVs), with 301 SNVs and 131 SNVs in 37MB respectively; using MSIsensor, patient 1 was identified as MSI-H (5/5 markers positive) and patient 2 harbored a *POLE* p.G541E mutation of unknown significance, but her primary tumor was MSS.

### 3.2. ctDNA Can Detect EC Recurrence and Progression Earlier than Scans

**Figure 1:**
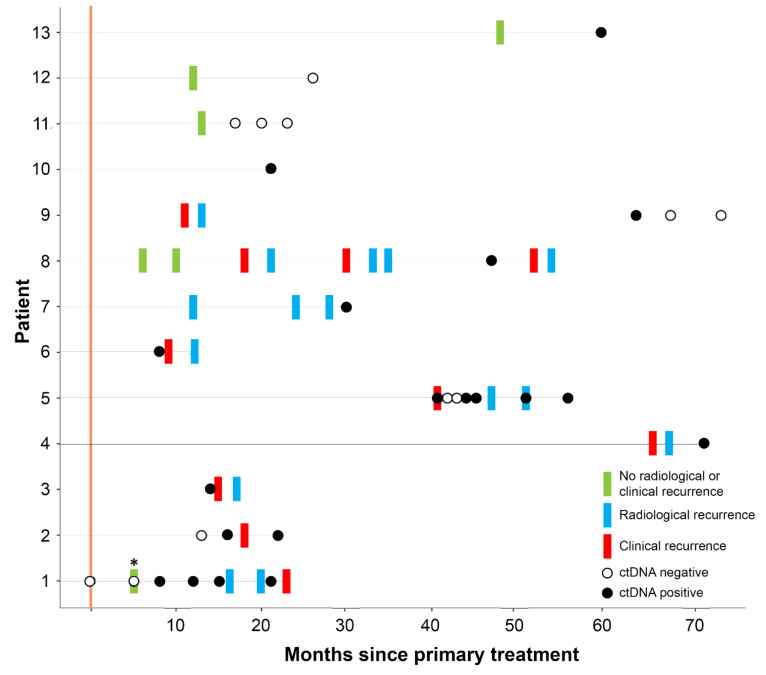
Summary of each patient’s results of clinical imaging and results of serial samples in terms of detection of circulating tumor DNA (ctDNA). Asterisks above sample for patient 1 indicates mutation was present in the Integrated Genomics Viewer (IGV) but not called due to high strand bias.

The median lead time of ctDNA over radiological imaging or clinical recurrence in patients for whom we had sample available prior to recurrence or progression was 2.5 months (*n* = 6, range 1–8 months) (Figure 1). ctDNA was detected in all four patients (100%) who were diagnosed with stage I disease at diagnosis and who subsequently recurred (Figure 1, Table S2). For patient 1, the lead time of ctDNA (determined through detection of *MUC4* p.H2306P mutation and MSI markers) over radiological imaging was 8 months, and there were 16 months between the first positive ctDNA sample and the patient developing symptoms (Figure 1). Two of the stage I patients (cases 1 and 2) had serial samples analyzed, and disease progression was confirmed with increasing ctDNA levels, in concordance with both clinical and imaging progression (case 2—*MUC4* p.A2313V and MSI markers). ctDNA was detected in the four patients (100%—patients 5–8) who were diagnosed with stage IV disease and had progressive disease on imaging (Figure 1, Table S2). Mutations detected in these patients and variant allele fractions are given in Table S2.

### 3.3. ctDNA Accurately Reflects EC Disease Kinetics during Treatment

**Figure 2:**
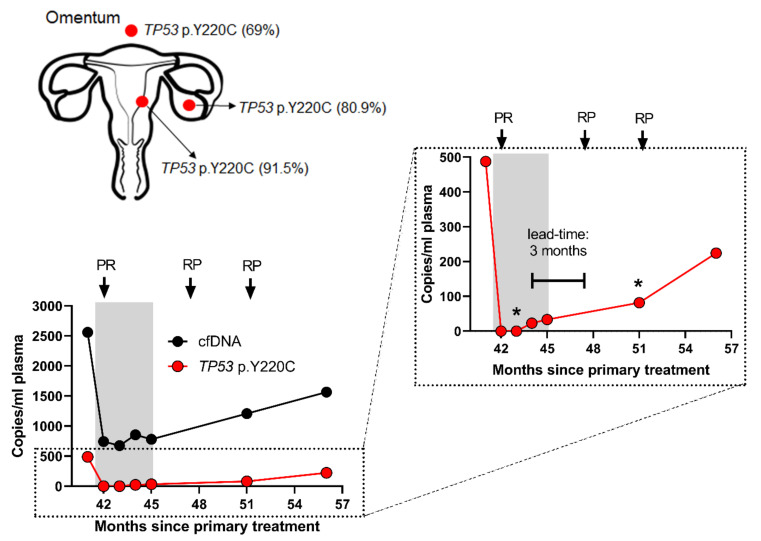
Multiregional sampling from patient 5, alongside the respective variant allele frequencies of the major driver mutation, *TP53* p.Y220C, identified in the primary tumor sample (located in the endometrium), local metastatic lesion (located in the ovary) and the distal metastatic lesion (located in the omentum). The levels of cell-free DNA (cfDNA) and ctDNA in pre-treatment and post-treatment plasma samples are shown in the accompanying left-hand graph, clearly demonstrating a rise in ctDNA levels 3 months prior to radiological relapse (right-hand). * samples analyzed exclusively by digital droplet PCR (ddPCR). Grey shaded area indicates treatment with carboplatin and paclitaxel. PR = partial response; RP = radiological progression.

ctDNA levels accurately mirrored the radiological response of a patient undergoing chemotherapy treatment for recurrent EC, with a clonal *TP53* p.Y220C mutation becoming undetectable during treatment, before rising again prior to radiological progression (Figure 2). An additional two patients (cases 9 and 11) diagnosed with stage I EC received radiotherapy, one for a vaginal vault recurrence, and the other as primary treatment, respectively. For case 11, a single *PIK3CA* p.G106V mutation detected in her primary tumor was not detected in three plasma samples following primary radical radiotherapy, in keeping with clinical findings of complete response (Figure 1). *PIK3CA* p.H1047R, *MET* p.T1010I, *FGFR2* p.S252W, and *KRAS* p.G12A mutations were detected in the first plasma DNA following treatment of the vault recurrence (case 9—Table S2); however, it became negative with time and remained negative, in keeping with the clinical and radiological findings of complete resolution of the recurrence and no ongoing disease (Figure 1).

### 3.4. Follow-Up of High-Risk EC Cases

In the three cases with high-risk EC at diagnosis (stage III non-endometrioid histology), ctDNA was detected in only one case (Patient 13—*TP53* p.S241F. Figure 1, Table S2). All patients were alive at the consensus date, with no clinical or radiological sign of recurrence and continue to be followed-up.

### 3.5. Longitudinal ctDNA Can Detect Acquired Microsatellite Instability

**Figure 3:**
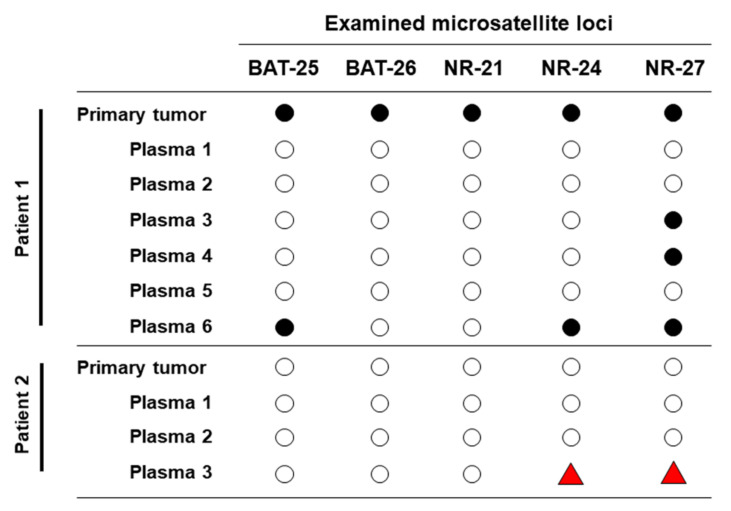
Microsatellite status in the primary tumors and longitudinal plasma samples of patients 1 (high microsatellite instability (MSI-H)) and 2 (MSS).
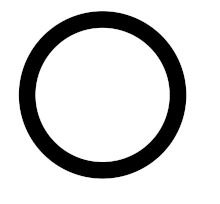
 = alleilic shift not detected;
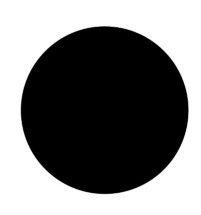
 = alleilic shift detected; 
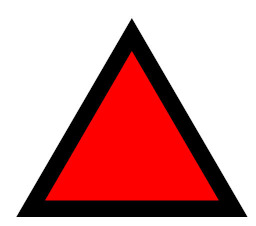
 = alleilic shift has changed from negative to positive.

Only one patient’s tumor (patient 1) was classified as MSI-H, with all five markers displaying microsatellite instability, which was confirmed in ctDNA (3/5 markers positive—BAT25, NR-24 and NR-27) (Figure 3). In addition, ctDNA was able to identify the acquisition of microsatellite instability, in a case (patient 2) whose primary tumor was microsatellite stable (MSS). The patient had received adjuvant chemotherapy but had subsequently recurred. Longitudinal samples revealed the acquisition of MSI-H status, with two of the five markers (NR-24 and NR-27) displaying MSI in the last plasma sample (Figure 3, Table S2). 

### 3.6. ctDNA is Reflective of EC Tumor Heterogeneity and Evolution

Analysis of the primary, metastatic and recurrent tumor biopsies and matched plasma DNA for two cases (cases 5 and 9) demonstrated that mutations identified in biopsies from different locations were present in the ctDNA, but that additional mutations could also be identified. Truncal *TP53* p.Y220C (case 5) and *PIK3CA* p.H1047R (case 9) mutations were identified in primary and recurrent tumors but additional mutations, *MET* p.T1010I, *FGFR2* p.S252W, and *KRAS* p.G12A, were detected in the ctDNA at recurrence that were not present in the tumor samples (Table S2).

## 4. Discussion

Despite the limited numbers in our cohort, we have shown the potential of ctDNA as a highly sensitive biomarker for identifying endometrial cancer recurrence in different histological subtypes and grades, and has a lead time of up to 16 months over patient reported symptoms and up to 8 months compared to imaging. In addition, we have suggested that ctDNA levels can accurately reflect disease activity, with earlier identification of response/progression than on CT imaging. We also report the first case of ctDNA identifying the acquisition of microsatellite instability in a case of EC recurrence, an increasingly important stratifying feature for determining optimum patient treatment.

Our results are in keeping with studies published in other tumor types, and support the clinical utility of ctDNA in early detection of recurrence in endometrial cancer [35] and treatment stratification in other solid tumors [6,30,51]. Previous studies have shown that while ctDNA can be detected in patients with more aggressive histologies (such as serous and carcinosarcomas) [35], detection of ctDNA in patients with endometrioid histologies (i.e., the vast majority of EC patients) is more challenging [24,26]. The use of different technologies for ctDNA detection has given further insight into the optimum methods that can be taken forward for use in a clinical trial determining the potential for this new development on patient management, especially those with endometrioid histologies. We have shown a benefit in using WES to profile the tumor tissue and tracking mutations with a customized targeted cfDNA panel relative to using a driver gene approach, which did not identify any mutations in four tumor samples, equating to approximately one third of our cohort who otherwise would not have been monitored. Additionally, the customized targeted panel can give greater informative value in monitoring of tumor evolution, identifying somatic variants not present in the primary tumor tissue. This further highlights the beneficial value of using a targeted customized panel approach over a driver gene approach, as it may not elucidate the evolving genetic proportions as accurately as the personalized approach.

At present there is no EC specific blood tumor marker to monitor for disease recurrence, unlike CA125 and ovarian cancer. Our results have shown the potential of ctDNA to monitor patients undergoing treatment, as compared to waiting for radiological response. Parkinson et al., reported that chemotherapy response for the treatment of high-grade serous ovarian cancer was detected earlier with ctDNA, as compared to CA125, with a nadir of 37 days compared to 84 days respectively, and ctDNA was more prognostic than CT imaging [52]. Resistance to first line chemotherapy for EC is known to be a greater issue than with ovarian cancer [53], and, should our results be replicated in a larger population, it opens up the potential for ctDNA identification of non-efficacious treatments at an earlier time point, rather than waiting for imaging assessment of disease response.

Our results have also shown an additional benefit of longitudinal monitoring of patients using personalized assays containing MSI markers with the discovery of acquired MSI-H status through serial plasma sampling. ctDNA analysis has been shown to detect MSI [54], reflect tumor heterogeneity [55], and track tumor evolution [29]. Acquired MSI-H status has previously been shown in prostate cancer [56], however, to our knowledge, this is the first report of acquired MSI-H status in EC. The presence of a MMR gene deficiency is known to be positively associated with response to PD-1 inhibitors [57] and the approval of Pembroluzumab has the potential to benefit up to 30% of advanced EC patients [58,59]. This has led to the recommendation that all EC patients undergoing MSI testing [60]. Indeed, in the relapse setting, the use of Pembrolizumab has been modeled to be cost-effective in MSI-H patients, as compared to single agent pegylated liposomal doxorubicin or bevacizumab [61]. The identification of acquired MSI mutations in relapsed EC highlights another population of patients who could potentially benefit from a PD-1 inhibitor, but who would not be eligible if their MSI status was determined from their primary tumor alone. However, it is prudent to note that our study only detected acquired MSI-H status in one patient and much larger studies highlighting this are needed, before significant conclusions can be made regarding its utility. The ability of ctDNA to detect such tumor evolutions through a test with high patient acceptability [62], and without the need for invasive biopsies or surgery, could further support the development of personalized patient management and, hopefully, improved patient outcomes.

## 5. Conclusions

In conclusion, ctDNA monitoring in EC has the potential to accurately monitor the activity of endometrial cancer and diagnose recurrence. However, caution is suggested, since further work in a larger cohort is needed to determine the lead time and its ability to identify local, as well as distant, recurrent disease.

## Figures and Tables

**Table 1:** Tumor characteristics and outcome of study cohort (*n* = 13).

Patient	Histology	Stage at Diagnosis	Recurrence	Site of Recurrence	Outcome
1	Endometrioid G1	IB	Yes	Pelvic	Alive with disease
2	Endometrioid G3	IB	Yes	Distant	Died with disease
3	Serous	IA	Yes	Distant	Alive with disease
4	Endometrioid G1	IB	Yes	Pelvic, distant	Alive with disease
5	Endometrioid G3	IVB	Yes	Distant	Alive with disease
6	Endometrioid G2	IVB	Yes	Distant	Died with disease
7	Endometrioid G1	IVB	Yes	Distant	Alive with disease
8	Carcinosarcoma + Serous	IVB	Yes	Pelvic, distant	Alive with disease
9	Endometrioid G1	IA	Yes	Vaginal vault	Alive no disease
10	Endometrioid G1	I	No	NA	Died no disease
11	Serous	IIIC1	No	NA	Alive no disease
12	Clear Cell	IIIB	No	NA	Alive no disease
13	Carcinosarcoma	IIIA	No	NA	Alive no disease

## Supplementary Materials

The following are available online at https://www.mdpi.com/2072-6694/12/8/2231/s1, Table S1: Genomic coordinates for personalised ctDNA panel and target mutations derived from whole exome sequencing of the primary tumor, Table S2: The somatic mutations identified in the matched tumor-normal patient samples analyzed (*n* = 13).

## Preprint

The results of this work were deposited as a preprint in MedRxiV (https://www.medrxiv.org/content/10.1101/2020.03.04.20030908v2).
