# Supplementary material for: Utility of Circulating Tumor DNA for Detection and Monitoring of Endometrial Cancer Recurrence and Progression
Source: Cancers (Basel). 2020 Aug 10;12(8):2231. doi: 10.3390/cancers12082231 (PMC7463944; doi:10.3390/cancers12082231)
Supplement: Supplementary file 1 [file cancers-12-02231-s001.pdf]

Supplementary Material: Utility of Circulating Tumor DNA for Detection and Monitoring of Endometrial Cancer Recurrence and Progression

Esther L. Moss, Diviya N. Gorsia, Anna Collins, Pavandeep Sandhu, Nalini Foreman, Anu Gore, Joey Wood, Christopher Kent, Lee Silcock, David S. Guttery

Table S1. Genomic coordinates for personalised ctDNA panel and target mutations derived from whole exome sequencing of the primary tumor.

Chromosome	Start	End	Target
chr1	37984113	37984293	SF3A3 p.C92S_1,SF3A3 p.C92S_2
chr2	47414258	47414558	BAT-26_1,BAT-26_2,BAT-26_3,BAT-26_4
chr2	95183472	95183653	NR-24_1,NR-24_2,NR-24_3,NR-24_4
chr3	195783832	195784012	MUC4 p.P2562S_MUC4 p.S2544P_1,MUC4 p.P2562S_MUC4 p.S2544P_2
chr3	195784551	195784731	MUC4 p.A2313V_1,MUC4 p.A2313V_2
chr4	54731896	54732196	BAT-25_1,BAT-25_2,BAT-25_3,BAT-25_4
chr6	32221013	32221193	NOTCH4 p.G225R_1,NOTCH4 p.G225R_2
chr7	100952460	100952640	MUC3A p.P258S_1,MUC3A p.P258S_2
chr7	100953384	100953564	MUC3A p.H563Q_MUC3Ap.S568Y_1,MUC3A p.H563Q_MUC3Ap.S568Y_2
chr9	8375930	8376110	PTPRD p.E1119K_1,PTPRD p.E1119K_2
chr10	87933057	87933237	PTEN p.R130Q_PTEN p.R130L_1,PTEN p.R130Q_PTEN p.R130L_2
chr10	121520072	121520252	FGFR2 p.S252W_1,FGFR2 p.S252W_2
chr11	1017234	1017414	MUC6 p.Y1826D_1,MUC6 p.Y1826D_2
chr11	1017562	1017764	MUC6 p.P1716S_1,MUC6 p.P1716S_2,MUC6 p.P1716S_3
chr11	14358713	14358893	RRAS p.G23C_1,RRAS p.G23C_2
chr11	46898858	46899038	LRP4 p.D211H_1,LRP4 p.D211H_2
chr11	102322679	102322919	NR-27_1,NR-27_2,NR-27_3
chr13	48459617	48459797	RB1 p.R661W_1,RB1 p.R661W_2
chr14	23182997	23183297	NR-21_1,NR-21_2,NR-21_3,NR-21_4
chr17	43091512	43091692	BRCA1 p.T1263S_1,BRCA1 p.T1263S_2
chr19	8888681	8888861	MUC16 p.S13577G_MUC16 p.H13576N_1,MUC16 p.S13577G_MUC16 p.H13576N_2
chr19	52212627	52212807	PPP2R1A p.P179R_1,PPP2R1A p.P179R_2

Table S2. The somatic mutations identified in the matched tumour-normal patient samples analysed (n = 13).

Patient ID	ctDNA Detected?	Mutations Detected in Tumor	%VAF in Tumour	Mutation(s) detected in ctDNA (Any Timepoint)	Timepoint Detected (%VAF)	Total Timepoints
1 ^Δ^	Y *	*PTEN* p.R130Q	14.3	*MUC4* p.H2306P	3 (9.57%), 5 (7.34%)	6
		MUC16 p.H13577G	14.6	MSI markers	3 (1/5 markers), 4 (1/5 markers), 6 (3/5 MSI markers)	
		MUC4 p.S2544P	26.2			
		MSI markers	5/5 detected			
2 ^Δ^	Y	*MUC4* p.A2313V	16	*MUC4* p.A2313V	2 (42.3%), 3 (54.35%)	3
		PTEN p.R130L	19.3	MSI markers	3 (2/5 MSI markers)	
		MSI markers	None			
3 ^Δ^	Y *	*PTPRD* p.E1119K	20.3	*MUC4* p.H2290L	1 (9.48%%)	1
		MUC6 p.P1716S	20.7	*MUC4* p.A2313V	1 (19.48%)	
		SF3A3 p.C92S	21.2			
		MUC4 p.P2562S	24.8			
		PPP2R1A p.P179R	27.3			
		MUC6 p.Y1826D	37.5			
		LRP4 p.D211H	55.2			
		RRAS p.G23C	58.3			
4	Y±	*PIK3CA* p.H1047L	18.53	*PIK3CA* p.E545A	1 (0.16%)	1
		KRAS p.G12A	20.67	*PIK3CA* p.H1047L	1 (2.19%)	
				KRAS p.G12A	1 (2.12%)	
				CTNNB1 p.T41A	1 (2.29%)	
5	Y	*TP53* p.Y220C (primary tumor and 2 metastatic sites)	91.59 (primary tumour), 80.91 (ovary), 69.63 (omentum)	*TP53* p.Y220C	1 (19.05%), 4 (2.72%), 5 (4.3%), 6 (6.76%), 7 (14.3%)	7
				AKT p.E17K	4 (3.05%)	
6	Y ±	*PIK3CA* p.H1047R	59.65	*PIK3CA* p.H1047R	1 (1.18%)	1
		PTEN p.Y155C	33.58	*ESR1* p.D538G	1 (2.75%)	
7	Y	*TP53* p.R273C	85.39	*TP53* p.R273C	1 (1.53%)	1
8 ^Δ^	Y ±	*BRCA1* p.T1263S	14.9	*MUC4* p.A2313V	1 (10.56%)	1
		MUC16 p.H13576N	15.7	*NOTCH4* p.G225R	1 (50.59%)	
		MUC3A p.H563Q	20.2			
		RB1 p.R661W	21.4			
		NOTCH4 p.G225R	21.5			

9	Y ±	*PIK3CA* p.H1047R (primary and relapse)	27.94 (primary tumour), 35.33 (relapse biopsy)	*PIK3CA* p.H1047R	1 (3.03%)	3
				MET p.T1010I	1 (16.71%)	
				FGFR2 p.S252W	1 (2.15%)	
				KRAS p.G12A	1 (1.89%)	
10	Y *	*KRAS* p.G12A	22.78	*PIK3CA* p.E545A	1 (0.43%)	1
		PIK3CA p.G1049R	12.6			
11	N	*PIK3CA* p.G106V	35.14	None detected	-	3
12	N	*PIK3CA* p.R88Q	18.32	None detected (*KRAS* p.G12D)	-	1
		KRAS p.G12D	23.99			
13	Y *	*PIK3CA* p.E545A	38.09	TP53 p.S241F	1 (0.28%)	1
		TP53 p.C242F	40			

[Δ] Patient's whose tumor underwent whole exome sequencing. * ctDNA positive patient where mutation in ctDNA did not correlate with tumor. [±] Additional mutations detected in ctDNA. Note that mutations in bold were validated by ddPCR. For patient 1, mutation was also detected at timepoint 2 but not called due to poor sequencing over the locus.
